# Determination of oleanolic acid and ursolic acid contents in *Ziziphora clinopodioides* Lam. by HPLC method

**DOI:** 10.4103/0973-1296.62898

**Published:** 2010-05-05

**Authors:** Shuge Tian, Yang Shi, Qian Yu, Halmurat Upur

**Affiliations:** *Xinjiang Key Laboratory of Famous Prescription and Science of Formulas, Urumqi-830011, Xinjiang, College of TCM, Xinjiang Medical University, Urumqi-830011, Xinjiang, China*

**Keywords:** HPLC, oleanolic acid, ursolic acid, *Ziziphora clinopodioides* Lam

## Abstract

A simple, precise, rapid and accurate, binary-phase high performance liquid chromatographic method has been developed for the determination of oleanolic acid and ursolic acid contents in the *Ziziphora clinopodioides* Lam. with short run time. Chromatographic separation is achieved by using HPLC system consisting of a Shimadzu LC-6AD and Kromasil C_18_ column (150 × 4.6 mm, 10 μm, with pre-column), the mobile phase consists of methanol and 0.03 M phosphate buffer (pH = 3, 90:10). Detection wavelength is 214 nm. The speed of flow is 0.5 ml/min. The specimen handing quantity is 10 μl. The oleanolic acid's linearity range is 0.4 ~ 1.2 mg/ml (r = 0.9996). The ursolic acid's linearity range is 0.6 ~ 1.8 mg/ml (r = 0.9996), and the linear relationship is accurate. The average recovery (*n* = 6) of oleanolic acid is 99.5% (RSD = 1.19%) and ursolic acid is 102.3% (RSD = 1.25%). The content of oleanolic acid and ursolic acid in *Ziziphora clinopodioides* are 0.76 mg/g and 1.176 mg/g, respectively. The developed HPLC method can therefore be applied to both *in vitro* studies of oleanolic acid and ursolic acid formulations as well as drug estimation in biological samples.

## INTRODUCTION

Oleanolic acid and ursolic acid are the common constituents of plants. These two triterpenes may occur as aglycones of saponins and free acids. Reports on their wide-ranging occurrence in one of the most important groups of medicinal plants, the family Lamiaceae, usually also describe the isolation of free oleanolic acid and ursolic acid besides other compounds.[1] Both oleanolic acid and ursolic acid have many important pharmacological effects, which are rather similar because of the closeness of their chemical structures. The literature furnishes numerous data on their anti-inflammatory,[2] hepatoprotective,[3] antitumor,[4] anti-HIV,[5] antimicrobial,[6] antifungal,[7] gastroprotective,[8] hypoglycemic[9] and antihyperlipidemic[10] properties. They are relatively non-toxic and have been used in cosmetics and some health products.[3] Unfortunately, insufficient information is available concerning the distributions of oleanolic acid and ursolic acid in the *Ziziphora clinopodioides* Lam, because the published quantitative investigations generally extended to only a few species, and mainly qualitative data are to be found only with the presence of these compounds.

*Ziziphora clinopodioide Lam* is a traditional Uygur medicinal plant, which is a semi-perennial shrub-like plant that grows on low hills, grassland and arid slopes and is widely distributed in China, Mongolia, Turkey, Kazakhstan and Kyrgyzstan.[11] It is mainly used for the treatment of heart disease, high blood pressure, asthma hyperhidrosis, palpitation insomnia, edema, cough, bronchitis, lung abscess and other diseases. The results of animal experiments show that it can significantly prolong the survival time of hypoxic-mouse model for its good effect against myocardial ischemia and hypoxia. So far, the research on the *Ziziphora clinopodioides* focuses on the chemical constituents of volatile oil, as well as antibacterial activities. In preliminary work, we have also studied the stability of the volatile oil,[12] and the different parts of polar solvent extraction in antioxidant activities, but HPLC method used for the determination of oleanolic acid and ursolic acid content has not been reported.

## MATERIALS AND METHODS

### Plant material and reagents

The plant (whole plant) used for the present study is collected from Wulabo of Urumqi, Xinjiang province. Plant materials are further identified by Yonghe Li, a Pharmacist from Chinese medicine hospital of Xinjiang. Oleanolic acid and ursolic acid are obtained from National Institute for The Control of Pharmaceutical and Biological Products, 110709-200304110742-200516. HPLC grade Methanol supplied by USA, Fisher Scientific, 201-796-7100. Potassium phosphate monobasic of analytical grade is obtained from Tianjin, Tianxin chemical reagents company. All reagent solutions and buffers are prepared with water from Millipore Q3 ultra-pure water system (Millipore, USA).

### Instrumentation and chromatographic conditions

The HPLC system consists of a Shimadzu LC-6AD, SPD-20AVP variable wavelength UV detector and Shimadzu CBM-20A station for data analysis. The analytical column is Kromasil C_18_ column (4.6 × 150 mm, 10 μm, with precolumn). The mobile phase is methanol-0.03M phosphate buffer (pH = 3, 90:10). The flow rate is 0.5 ml/min. The effluent is monitored for UV absorption at 214 nm. The injection volume is 10 μl. All separations are performed at ambient temperature.

### Sample preparation

Powder samples (10.0 g) are extracted by methanol (100 ml) for 30 min ultrasonic extraction, filtered, dried in water bath, dissolved with methanol and transferred to a 10 ml measuring flask, and finally, have the volume to the calibration using methanol and shaken evenly before filtering in a 0.45 μm membrane filter.

### Standard solution preparation

A standard solution containing 2.0 mg/ml of oleanolic acid and 3.0 mg/ml of ursolic acid is prepared by dissolving in methanol.

## RESULTS

### Optimization of the chromatographic conditions

**Figure 1:**
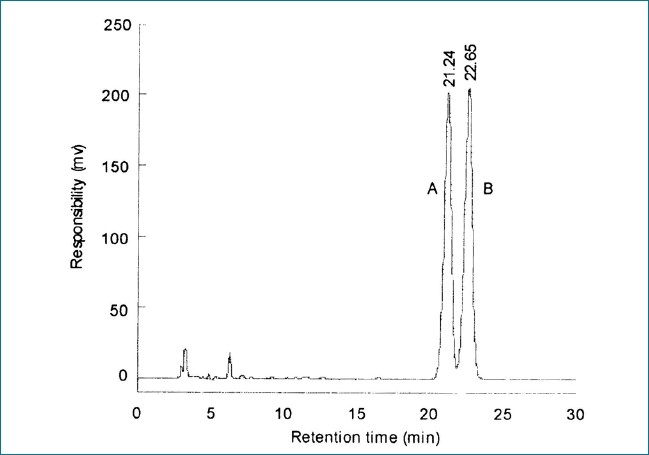
Standard solution chromatogram (A: Oleanolic acid, B: Ursolic acid)

**Figure 2:**
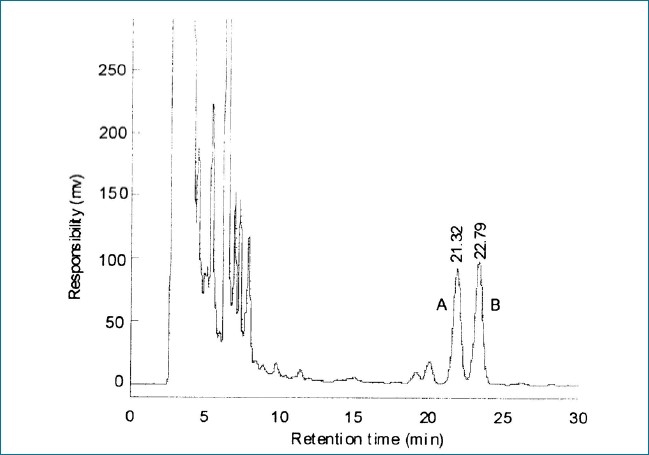
*Ziziphora clinopodioides* sample solution chromatogram (A: Oleanolic acid, B: Ursolic acid)

LC parameters are optimized by investigating the influence of the mobile phase, column temperature and detection wavelength. The initial separation is carried out on an Agilent Zorbax Extend C_18_ reserved-phase column with mixtures of methanol and water while the mobile phase with a gradient elution method. Because the peak shapes are unsatisfactory, a small amount of phosphate buffer is added to the mobile phase in order to suppress the ionization of these compounds, sharpen peak shapes and improve analytical sensitivity and resolution. The optimum mobile phase was composed of methanol and phosphate buffer (pH = 3, 90:10), with a flow rate of 0.5 ml/min, and detection wavelength of 214 nm. Retention time for oleanolic acid and ursolic acid are 21.93 and 23.38 min, respectively. The results are shown in Figures 1 and 2. An HPLC chromatograph of oleanolic acid and ursolic acid in standard solution is shown in Figure 1, an HPLC chromatograph of oleanolic acid and ursolic acid in sample solution is given in Figure 2.

### Specification curve

Oleanolic acid standard curve: *y* = 5138012.5x - 110328.8 (x is the concentration of oleanolic acid and y is the peak area integral value of oleanolic acid). The oleanolic acid's linearity range was 0.4-1.2 mg/ml, with a mean correlation coefficient *r* = 0.9996.

Ursolic acid standard curve: y = 3693315.3x - 113635.2 (x is the concentration of ursolic acid and y is the peak area integral value of ursolic acid). Ursolic acid's linearity range was 0.6-1.8 mg/ml, with a mean correlation coefficient *r* = 0.9996.

### Precision

Take the oleanolic acid and the ursolic acid, mix with 10 μl standard comparison solution, and determine according to the above chromatographic condition. Handle the specimen six times. Determine the oleanolic acid and the ursolic acid peak area integral values. RSD% is 1.53 and 1.13%, respectively. The results obtained confirm a good precision of the method developed. The results are shown in Table 1.

**Table 1:** Precision conclusion

No	Oleanolic acid sample peak area integral value	Ursolic acid sample peak area integral value
1	4436286	4842616	
2	4602612	4962482
3	4581736	4947798
4	4624223	4996164
5	4615594	4993141
6	4596357	4949598
RSD (%)	1.53	1.13

### Stability

Take the identical sample to supply the test solution for 0, 2, 4, 8, 24 h, respectively, and determine the oleanolic acid and the ursolic acid peak area integral values. RSD % is 1.40 and 1.58% respectively. The experimental results indicate that samples are stable in 24 h. The results are shown in Table 2.

**Table 2:** Stability conclusion

Time (h)	Oleanolic acid peak area integral value	Ursolic acid peak area integral value
0	3884202	4233366
2	3828496	4151307
4	3841187	4125055
6	3794021	4089668
8	3741026	4063406
RSD (%)	1.40	1.58

### Reproducibility

Take *Ziziphora clinopodioides* Lam powder 10.0 g, according to “2.2” strip by the same method. Determine the oleanolic acid and the ursolic acid peak area integral values according to the above chromatograph condition, RSD % is 1.86% and 1.62 % respectively. The experiment results indicate that the accuracy is good. The results are shown in Table 3.

**Table 3:** Reproducibility conclusion

No.	Oleanolic acid peak area integral value	Ursolic acid peak area integral vaule
1	3840722	4294788
2	3781915	4203736
3	3692671	4176719
4	3748147	4175203
5	3895012	4227854
6	3798748	4308687
RSD (%)	1.86	1.62

### Recovery

Take 5.0 g *Ziziphora clinopodioides* Lam of the known content for each group and add the 3.80 mg oleanolic acid standard solution and 5.92 mg ursolic acid standard solution and calculate the spotting recovery rate. According to “2.2” strip by the same method and computation content using standard curve method, the average recovery (n = 6) of oleanolic acid is 99.5% (RSD = 1.19%) and of ursolic acid is 102.3% (RSD = 1.25%). The results are shown in Tables 4 and 5.

**Table 4:** Recovery rate of oleanolic acid conclusion

Sampling quantity(g)	Sample content A(mg)	Addition B(mg)	Determination C(mg)	Recovery(%)	Average(%)	RSD(%)
5.00	3.80	3.80	7.64	101.1		
5.00	3.80	3.80	7.59	99.7		
5.00	3.80	3.80	7.51	97.6	99.5	1.19
5.00	3.80	3.80	7.58	99.5		
5.00	3.80	3.80	7.56	98.9		
5.00	3.80	3.80	7.61	100.2		

Recovery rate = [(C-A)/B]×100%

**Table 5:** Recovery rate of ursolic acid conclusion

Sampling quantity(g)	Sample content A(mg)	Addition B (mg)	Determination C(mg)	Recovery (%)	Average (%)	RSD (%)
5.00	5.88	5.92	12.01	103.5		
5.00	5.88	5.92	11.93	102.1		
5.00	5.88	5.92	11.83	100.5	102.3	1.25
5.00	5.88	5.92	11.95	102.5		
5.00	5.88	5.92	12.03	103.8		
5.00	5.88	5.92	11.87	101.2		

Recovery rate = [(C-A)/B]×100%

### Quantification of oleanolic acid and ursolic acid in sample

The standard addition and recovery experiments are conducted to determine the accuracy of the present method for the quantification of oleanolic acid and that of ursolic acid in *Ziziphora clinopodioides* Lam samples. The content of oleanolic acid and ursolic acid in *Ziziphora clinopodioides* Lam are 0.76 mg/g and 1.176 mg/g, respectively.

## DISCUSSION

A simple and sensitive HPLC method is developed for the determination of oleanolic acid and ursolic acid contents in *Ziziphora clinopodioides* Lam. This easy method provided excellent sensitivity, accuracy and precision, with relatively short retention time for both oleanolic acid and ursolic acid. This developed HPLC method can therefore be applied to both *in vitro* studies of oleanolic acid and ursolic acid formulations as well as drug estimation in biological samples.
